# Assessment of vaccine wastage in South Sudan

**DOI:** 10.11604/pamj.2021.40.114.28373

**Published:** 2021-10-22

**Authors:** James Bol, Nathan Atem Anyuon, Evans Nyasimi Mokaya

**Affiliations:** 1 Evans Nyasimi Mokaya, African Field Epidemiology Network, Juba, South Sudan

**Keywords:** Vaccine wastage, South Sudan, vaccination, supply chain

## Abstract

**Introduction:**

vaccine utilization monitoring provides valuable information for practical forecasting and formulation of strategies to reduce avoidable wastage. This monitoring is weak at county and health facility levels in South Sudan. Lack of national wastage rates could result in inaccurate forecasting, leading to vaccine shortages or overstocking and expiration of vaccines at the subnational and service delivery points. As the country gears to introduce relatively expensive vaccines such as rotavirus and pneumococcal vaccines, a robust vaccine utilization monitoring system must be rolled out. This study provides the best possible estimates of vaccine wastage rates and the possible causes of the wastage.

**Methods:**

we conducted the study in 45 conveniently sampled health facilities across 9 of the ten states in South Sudan. Vaccine consumption data was prospectively collected to estimate vaccine wastage and the reason for the wastage of each vaccine type.

**Results:**

wastage of lyophilized vaccines, measles, and Bacillus Calmette–Guérin (BCG) ranged between 39.0-66.7% and 52.1-74.3%, respectively, mainly due to doses that were discarded 6 hours after the opening of the vial or at the end of the immunization session. Wastage of liquid vaccines Oral poliovirus vaccines (OPV), Penta, Inactivated polio vaccine (IPV), and Tetanus- diphtheria (Td) ranged between 24.4-49%, 15.5-43.4%, 25.3-57.9%, and 3.8-57.2%, respectively, mainly due to unusable VVM, expiry, unused doses at the end of outreach sessions, and vials without labels.

**Conclusion:**

wasted rates for all vaccines were higher than the indicative WHO wastage rates used in South Sudan to forecast national vaccine needs. Unopened vial wastage was high and needs immediate attention.

## Introduction

The landscape of vaccines used in low- and middle-income countries improved drastically during the decade of vaccines (2010-2020), narrowing the gap in accessibility to essential vaccines previously witnessed between these countries and the upper-income countries. During this period, low- and middle-income countries introduced relatively expensive vaccines such as DTP- hepB-Hib, rotavirus, and pneumococcal vaccines, increasing the cost per fully vaccinated child [1, 2]. Vaccines are generally expensive commodities that should be used efficiently to maximize the benefits and minimize wastage to ensure the immunization programs' sustainability. Despite the goodwill by global funding initiatives such as GAVI to solely or cost-share the procurement of vaccines used in developing countries; the vaccines' demand exceeds the available resources.

Despite WHOs reporting that over 50% of vaccines are wasted and advising countries to strengthen their systems to monitor wastage, progress has not been satisfactory [3, 4]. The current emphasis on increasing vaccination coverage and equity has made many countries not to focus attention on vaccine wastage monitoring and control. A significant number of GAVI supported countries do not have robust mechanisms to monitor vaccine wastage, especially at the service delivery levels [3]. The low drive for wastage monitoring and control could partly be premised on the evidence that too much focus on wastage could result in missed opportunities for vaccination [5-7]. The scales in this trade-off between wasting vaccines and increasing coverage are tilted in favour of the latter.

Vaccine wastage majorly occurs in two ways; 1) disposal of remaining doses of vaccines in an opened vial after the expiry of the recommended condition and duration that the vaccine can be used 2) unopened vials that are wasted due to exposure to excessive heat, freezing, expiry, pilferage, broken vials, and loss of labelling of the vaccines [8]. Granted that wastage from unused doses in opened vials of lyophilized vaccines is majorly unavoidable, the vaccine wastage caused by problems related to cold chain, pilferage, breakage, and expiry is mainly preventable. The factors that influence wastage of vaccines are broadly related to the vaccine characteristics, logistics, health worker behaviour, Economic Policy Institute (EPI) policy, and immunization practices [4].

The national vaccination program of South Sudan offers six vaccines against childhood diseases and maternal tetanus among women of childbearing age; BCG, OPV, Penta, IPV, Measles, and Tetanus- diphtheria (Td). Over the last ten years, Penta 3 coverage has remained suboptimal, with the highest coverage achieved in 2011 [9]. Based on WUENIC estimates, the highest coverages for Penta 1 and Penta 3 were 75% and 61% in 2011. Since then, the coverage declined to 51% and 49% in 2019 respectively. Between 2017 and 2019, the country did not experience vaccine stock-outs at the national and state levels [10]. However, there is no data on vaccines stock at county and health facility levels as a system to monitor vaccine utilization does not exist. Based on anecdotal reports, several facilities experienced stock-outs of vaccines, especially BCG, that remained unreported.

The monitoring of stock levels at the national level is done manually and electronically using the automated stock management tool (SMT) and Visibility for Vaccines (VIVA) tool. The SMT is yet to be rolled to the state and county levels. At state and county levels, manual (paper-based) monitoring is done by using the standard template that captures information on stock at the beginning of the month, stock receiving during the month, the current stock (physical counts) and the number of doses affected either through freezing, expiry, breakage or label detached captured in the tools. Due to inadequate numbers and skilled health workers, vaccine utilization and wastage monitoring are weak, especially at county and health facility levels. Poor forecasting has often resulted in either over-stocking or stock-outs of vaccines and supplies.

Data on vaccine utilization provide the program managers with useful information for practical forecasting and formulation of strategies to reduce the wastage of vaccines at all levels. The lack of evidence-based locally generated wastage rates coupled with lack of a robust system to monitor vaccine utilization at lower levels of service delivery could result in vaccine shortages or overstocking and expiration of vaccines at the subnational levels of service delivery. As the country envisages introducing relatively more expensive vaccines such as pneumococcal and rotavirus vaccines, regular monitoring of vaccine utilization is paramount.

This study presents wastage rates calculated from data collected in the sentinel facilities in South Sudan using the standard formulae; (N°. of doses wasted/N°. of doses issued) X 100), Vaccine Wastage Factor was calculated by using the formula 100/(100-vaccine wastage rate). The study also looks at the factors contributing to vaccine wastage at the service delivery level.

## Methods

**Study site:** the study was conducted in nine of the ten states in South Sudan. A total of 45 health facilities services participated in the study. The selected facilities consisted of hospitals, primary health care centres, and primary health care units that offered vaccination services. These facilities provide the entire spectrum of vaccines in the national vaccination program. While the BCG and OPV vaccine vials contain 20 doses, Penta, measles and Td vaccine vials have 10 doses. The IPV vial has 5 doses. BCG and measles are lyophilized vaccines, while the other four vaccines are presented as fully liquid vaccines. Penta, IPV and Td are administered intramuscularly while BCG, measles, and OPV are administered intradermally, subcutaneously, and orally, respectively

**Study design:** a descriptive cross-sectional study was employed. Data was prospectively collected from these facilities from January through August 2019.

**Study focus:** the study focus mainly on vaccine utilization monitoring and the reasons for vaccine wastage in the selected facilities.

**Sampling and data collection:** a non-random sample of 45 conveniently sampled health facilities offering vaccination services participated in the study. These facilities were in secure locations, easily accessible, in relatively urban settings, and regularly supported by the county and state managers. Routine immunization officers on training collected data on the starting stock levels every first working day of the month and data on new stock received during the month, vaccines issued to other facilities, remaining stock levels at the end of the month, and the number of children and pregnant women who were vaccinated in the month on the last working day of each month. This information was collected and transmitted to the lead investigator using kobo collect, an open phone-based data collection tool. The data was then transferred to Microsoft Excel for data cleaning before descriptive analysis was done using SPSS.

**Calculating vaccine wastage:** the number of used vaccines was calculated using the formulae ((total number of doses at the start of the month + the total number of doses received that month) - (no. of doses issued to other facilities + total doses remaining at the end of the month)). The number of wasted vaccines was calculated using the formulae (Used vaccines - the number of children or pregnant women who received the vaccine). We calculated the rate using the formula (number of doses wasted/number of doses used) x 100) while the wastage factor was calculated using the formulae (100/(100-wastage rate)).

**Statistical analysis:** statistical Package of Social Sciences (SPSS) for Windows version 25 (Inc., Chicago, IL, USA) was used for statistical analyses. Data are presented as means (range) for continuous variables and percentages for categorical variables. To explore the parameters associated with wastage rates, Analysis of Variance (ANOVA) test was used to compare wastage rates between types of facilities and a comparison between facilities that conducted outreaches versus those that did not conduct outreach services. Further, we computed binary logistic regression models to test the strength of association between wastage rate of vaccines (≤ indicative WHO rates used by the country versus > WHO indicative rates) wastage rate) as the dependent variable with the route of administration of the vaccine, formulation of the vaccine (fully liquid versus lyophilized), and the doses of vaccine in a single vial as independent variables. Odds ratio (OR) and 95% CI for the regression parameters are reported. Statistical significance was set at p<0.05.

## Results

**Figure 1:**
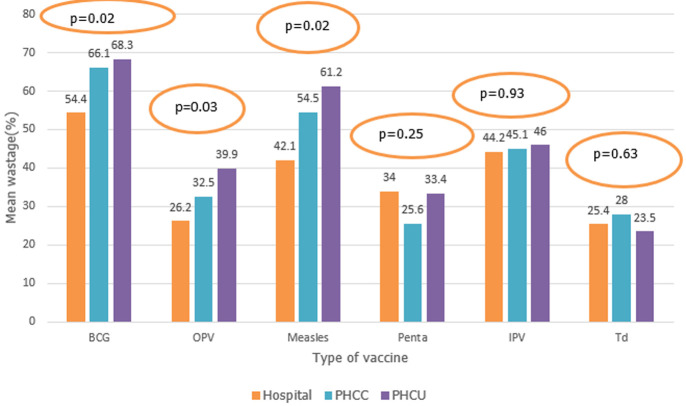
wastage rate by type of vaccine and type of facility

**Vaccine wastage rates:** the wastage rate differed by type of vaccine with BCG having the highest wastage at 64.4% (range = 52.1-74.3%) and Td having the lowest wastage at 26.9% (range 3.8% - 57.2%). Of note is that the IPV vaccine, which comes in a five-dose vial and is fully liquid, had a wastage rate of 45.1% (range = 25.3% - 57.9%). Measles vaccine that is a lyophilized vaccine had a wastage rate of 54.4% (range 39.0 - 66.7) while Penta and OPV had a wastage rate of 30% (range 15.5% - 43.4%) and 33.5% (24.4% - 49.0%), respectively. Although the wastage rates for the different vaccines varied among the states, as shown in Table 1, it is notable that all vaccines' wastage rates were significantly lower in Central Equatorial state (CES) compared to the other states. Figure 1 illustrates wastage rates by the type of facility. Overall, hospitals reported lower wastage rates of both lyophilized and fully liquid vaccines. The primary health care units (PHCU) wasted significantly higher BCG and measles vaccines than the hospitals and primary health care centres (PHCC). Also, the PHCU wastage a significantly higher OPV when compared to the hospitals. The wastage of other vaccines did not differ significantly among the levels.

**Table 1:** vaccine wastage by vaccine type and states in South Sudan

Vaccine type	Wastage	Central Equatoria State	Eastern Equatoria State	Lakes	Northern Bahr el Ghazal state	Unity	Upper Nile	Warrap	Western Bahr el Ghazal state	Western Equatoria State	South Sudan
**BCG**	**Mean vaccine wastage (%)**	56.3	66.1	52.1	71.3	56.5	64.5	74.3	64	69.4	64.4
	**Wastage factor**	2.3	3	2.1	3.5	2.3	2.8	3.9	2.8	3.3	2.8
**OPV**	**Mean vaccine wastage (%)**	25.9	34.6	49	24.4	33.8	43.9	36	34.3	40.7	33.5
	**Wastage factor**	1.4	1.5	2	1.3	1.5	1.8	1.6	1.5	1.7	1.5
**Measles**	**Mean vaccine wastage (%)**	39	60.7	65	66.7	41.6	62.4	61.8	58.8	58.7	54.4
	**Wastage factor**	1.6	2.5	2.9	3	1.7	2.7	2.6	2.4	2.4	2.2
**Penta**	**Mean vaccine wastage (%)**	15.5	35	31.4	24.7	37.7	43.4	38.7	32.7	32.5	30
	**Wastage factor**	1.2	1.5	1.5	1.3	1.6	1.8	1.6	1.5	1.5	1.4
**IPV**	**Mean vaccine wastage (%)**	25.3	49.2	57.9	51.4	53.9	53.8	50.3	52.9	52.5	45.1
	**Wastage factor**	1.3	2	2.4	2.1	2.2	2.2	2	2.1	2.1	1.8
**Td**	**Mean vaccine wastage (%)**	14.9	34.2	42.2	13.6	3.8	25	31.1	33.7	57.2	26.9
	**Wastage factor**	1.2	1.5	1.7	1.2	1	1.3	1.5	1.5	2.3	1.4

**Figure 2:**
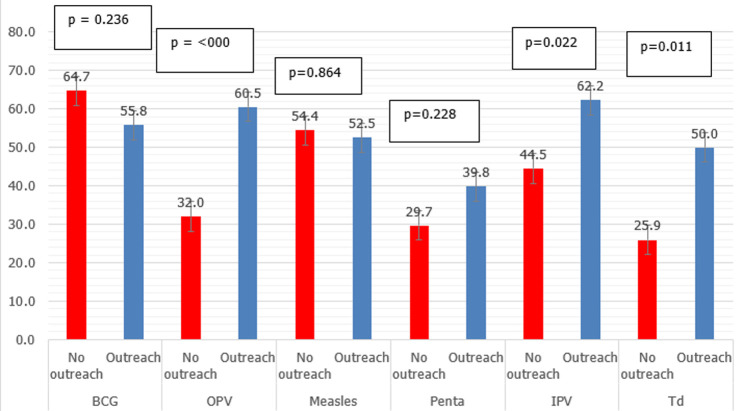
vaccine wastage by type of vaccine and service delivery strategy (fixed and outreach)

As illustrated in Figure 2, the wastage rates of IPV, Td, and OPV in facilities that conducted outreach and mobile services were significantly higher than facilities that only conducted fixed facility sessions. On the contrary, we noted no significant difference for BCG, measles, and Penta vaccines. After controlling for possible confounding factors, the lyophilized vaccines (BCG and measles) had 82% lower odds of having an acceptable wastage rate than the fully liquid vaccines used in the EPI program in South Sudan, as shown in (Table 2). The injectable vaccines and vaccines with 20 doses in a vial had a lower odd of an acceptable wastage rate than oral vaccines and vaccines with ten doses or five doses per vial, but the result was not significant.

**Table 2:** the relationship between vaccine wastage and key characteristics of the vaccines

Property of vaccine tested1	Adjusted odds ratio	P value
Route of administration	1.550 (0.826-2.909)	0.172
Formulation of vaccine	0.283 (0.181-0442)	<0.001
Doses in one vial of the vaccine	0.882 (0.556-1.398)	0.592

1Oral administration, fully liquid vaccines, ten doses vial are the comparators.

**Figure 3:**
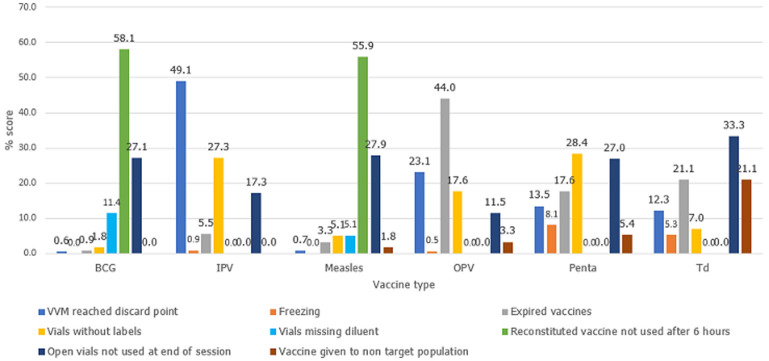
reasons for vaccine wastage by vaccine type

**Reasons for vaccine wastage:** Figure 3 presents the reasons behind the wastage by vaccine type. Wastage due to discarding of unused doses of BCG and measles, six hours after opening the vial, or at the end of the session accounted for the highest wastage, while freezing of IPV, Td, and Penta accounted for the lowest wastage.

Accumulated exposure to heat resulting in VVM changes contributed significantly to the wastage of the IPV vaccine. Compared to other vaccines, doses given to individuals outside the target population accounted for a significant wastage for the Td vaccine. On the contrary, not a single child above one year received IPV. Relatively more expired OPV vaccines were reported compared to other vaccines.

Accumulated exposure to heat resulting in VVM changes contributed significantly to the wastage of IPV vaccine. Compared to other vaccines, doses given to individuals outside the target population accounted for a significant wastage for the Td vaccine. On the contrary, not a single child above one year received IPV. Relatively more expired OPV vaccines were reported compared to other vaccines.

## Discussion

To the best of our knowledge, there are no prospective studies have been conducted in South Sudan to estimate the wastage rates of vaccines used in the EPI program. In the absence of national figures, South Sudan relies on indicative vaccine wastage rates by WHO to forecast and calculate the country's vaccine needs. Based on WHO projections, the wastage rate for freeze-dried vaccines (10-20 doses/vial) and liquid vaccines (10-20 dose/vials) are 50% and 25%, respectively [4]. This study shows that the wastage rate for all the vaccines was higher than the indicative WHO wastage rates.

Overall, BCG recorded the highest wastage among all vaccines. Our results are similar to other studies [11-13]. This could be attributed to the high number of doses per vial and the low numbers of eligible children per immunization session. Although the EPI policy in South Sudan discourages scheduling of EPI services on specific days, many health workers prefer to accumulate children and vaccinate them on a specific day in the week or month. This practice is common in other countries, such as in Nigeria [14]. Although scheduling BCG vaccination on specific days minimise vaccine wastage, it increases missed opportunities for vaccination [14]. Despite the Gambia not scheduling BCG vaccination, the wastage rate is relatively lower than our result, possibly because of the differences in the size of immunization sessions [13]. Our study's wastage rate is similar in India that uses a ten-dose vile compared to a 20-dose vial used in South Sudan [11, 12, 15].

The wastage rate for lyophilized vaccines was higher compared to the fully liquid vaccines. Similar results were reported by other studies [11, 13]. The relatively higher wastage rate could be attributed to the recommendation by WHO that all unused doses in opened vials of the two vaccines be discarded six hours after the opening of the vial or at the end of the immunization session, whichever comes first. Additionally, the number of eligible children per immunization session for BCG and measles is fewer than the Penta and OPV due to the number of doses required (3-4 doses for Penta and OPV versus single dose for measles and BCG). The wastage rates for lyophilized vaccines were statistically different between high and low volume facilities. This finding is consistent with other studies [16, 17] and supports the observation that the vaccination session's size was inversely associated with wastage of lyophilized vaccines.

Despite practicing the multi-dose vile policy (MDVP), the wastage rate for fully liquid vaccines in our study was higher than in other studies [13, 15]. The high overall wastage rate could be related to a high wastage of unopened vials due to suboptimal vaccine management practices. It is important to note that facilities that conducted outreach services recorded higher wastage for liquid vaccines than those that did not conduct outreach or mobile services. The reason for this wastage could be, as a rule, opened vials of all vaccines that were used in outreach or mobile clinic were discarded in order to safeguard the potency of the vaccines.

IPV wastage rate was relatively higher than the other liquid vaccines that can be used for 28 days if the MDVP conditions were strictly observed. Of note is that IPV vaccines recorded the highest proportion of vaccines that were wasted due to changes in the vaccine vial monitor. This could be explained by IPV having a more heat-sensitive VVM (VVM7) than the other vaccines with relatively stable VVM types. This would explain why measles and BCG recorded the lowest wastage rate due to changes in VVM.

Unopened vaccine wastage is needlessly high and needs immediate attention [4]. Expired vaccines (OPV, Penta, and Td), VVM changes (IPV), vials missing diluents (BCG and measles), and vials without labels accounted for a significant amount of wastage of vaccines. This could indicate suboptimal vaccine management practices and lack of vaccine visibility, especially at the service delivery level, contributing to vaccine wastage, as reported in the Effective Vaccine Management Assessment (South Sudan EVMA report) [18]. Expired OPV and Td vaccines could also result from the accumulation of vaccines used during the frequent supplemental immunization activities. Freezing of vaccines was never reported in any facility as a reason for vaccine wastage. Though this is a positive finding, there is a need to invest in building capacity of the vaccinators and their supervisors on fridge tags to validate this finding in the future. Currently, very few vaccinators and their immediate supervisors can correctly use the fridge tags in the field.

Vaccines given to children above one year and persons outside the target population accounted for the second least wastage. Surprisingly, save for Td given to trauma victims, very few doses of vaccines were given to children above one year even when the EPI policy recommends vaccination up to 23 months for all current childhood vaccines except BCG. The monthly and yearly EPI performance data confirms the low numbers of children above one year, and women of childbearing age receiving vaccines [19]. Vaccinating children up to 23 months would have a significant impact on vaccine-preventable diseases in South Sudan. Because the measles vaccine efficacy is significantly better among children above one year, ensuring that unvaccinated children between one and 23 months (as per the EPI policy) are offered the vaccines would significantly reduce the vaccine wastage and most importantly reduce the measles burden and the perennial measles outbreaks in the country [10]. There was no significant difference in the wastage rate between injectable and oral vaccines in our study. Similar results were reported in India [12]. The number of doses in a vial (alone) was not a significant influencer of vaccine wastage. A study in India reported contrary results on the relationship between vial size and the wastage rate [12]. A possible explanation for our results could be the high wastage of vaccines due to reasons other than the size of the immunization session. Of note is that while India used a ten dose vial of BCG, the wastage rate is similar to the wastage rate in our study and relatively higher compared to the wastage rate reported in the Gambia that uses a 20 dose BCG vile.

**Limitations:** the study was conducted among facilities that are easily accessible, secure, in relatively urban settings, and regularly supported by the county and state managers. Therefore, the wastage rates presented in this paper are the best possible rates and can, therefore, not be representative of the entire country.

## Conclusion

Vaccine wastage rates in our study are higher than the WHO indicative rates. Wasting of unopened vials significantly contributed to the overall wastage for all the vaccines. Whereas discarding unused doses of the liquid vaccines used in outreach services is a vital vaccine safety measure, it is a significant contributor to wastage of liquid vaccines in facilities that conduct regular outreaches. Vaccinating children aged between 12-23 months and non-gravid women of childbearing age is a less critical reason for “vaccine wastage” in South Sudan. This study recommends that vaccine wastage monitoring should be strengthened as an integral part of vaccine visibility at all levels. This could save significant resources if measures are put in place to minimize wastage without affecting vaccination coverage. On-the-job training on vaccine management, introduction of remote temperature monitoring, and supervision of vaccinators would go a long way in improving vaccine and cold chain management. The EPI program should prioritize health workers' sensitization to the benefits of vaccinating children aged between 12-23 months and non-pregnant women of childbearing age.

### What is known about this topic

- Disposal of remaining doses of vaccines in an opened vial after the expiry of the recommended condition and duration that the vaccine can be used is unavoidable. This study found that lyophilized vaccines (BCG and measles) had the highest rate of wastage because remaining doses in opened vials were discarded 6 hours after opening the vial or at the end of the immunization session;
- The average number of clients in a vaccination session is inversely related to vaccine wastage. Hospitals with high workload, compared to the primary health care units, had a lower vaccine wastage, especially for the multi dose lyophilized vaccines. This study proposes a mixed vial size approach for high and low volume facilities to minimize the wastage.

### What this study adds

- Vaccine wastage due to avoidable factors (unopened vial wastage) is high in South Sudan. VVM change, disposal of opened vial doses of fully liquid vaccines from outreach services, expiry of vaccines are significant contributors to vaccine wastage in South Sudan. This study recommends on-site mentorship of vaccinators on vaccine management at health facility level to minimize wastage of unopened vials;
- Despite being a fully liquid vaccine and a vial containing 5 doses, the IPV wastage is higher compared to the other fully liquid vaccines. This could be attributed to the VVM type on the IPV vial that is relatively heat sensitive. This study is suggesting using a higher wastage factor for IPV in fragile country with challenges with transportation and storage of vaccines to minimize vaccine stock outs.
